# Academy of Stomatology

**Published:** 1901-02

**Authors:** 

**Affiliations:** Academy of Stomatology


### ACADEMY OF STOMATOLOGY.

A REGULAR monthly meeting of the Academy of Stomatology of Philadelphia was held at the rooms of the Academy, 1731 Chestnut Street, on the evening of Tuesday, October 23, 1900, the President, Dr. J. T. Lippencott, in the chair.

A paper was read by Dr. William H. Trueman upon the subject "State Dental Examining Boards and their Questions."

(For Dr. Trueman's paper, see Vol. XXI., page 784.)

### DISCUSSION.

*Dr. Kirk.*—I regret exceedingly that a tone of partisanship seems to pervade the essay. In so far as the essayist has shown defects and evidences of occasional ignorance in the questions propounded by men who are appointed to examine into the qualifications of graduates, I think he has done a good work. To me he seems to have told but one side of the story. He assumes that a condition of antagonism exists between the teaching bodies and those who have the function of examining the product of their work; that the latter are of necessity partisan in their rulings, besides being ignorant. On the other hand, it is assumed that the teachers are men of high attainment, high motives, and apparently infallible. From my experience, I deny that either attitude really exists. The examination questions submitted by the faculties of the colleges may be criticised quite as freely as those of the boards. While these questions are defective, and many are ludicrous, I think the essayist has been hypercritical. There are a number of questions cited which, while awkward, at least convey the meaning of the examiners, and I think the average student ought to be able to answer a large proportion of them.

*Dr. Roberts.*—Fortunately, so far as I can see, Dr. Trueman has not selected any of my questions. There are several things I would like to criticise in Dr. Trueman's paper. I can say positively that there is no member of the Pennsylvania State Dental Examining Board but that welcomes honest and intelligent criticism, and by such he is helped in the formation of his questions. A great many of the questions that are given, and which are queerly worded, are evolved from questions previously asked, which are simple in their form but have not brought out the answers desired, showing that the students did not understand the meaning of the questions. These questions have been repeated in another form, to try to convey the meaning and bring out the knowledge. That is very frequently done. It is also very difficult to go into any given subject a second time very deeply without either repeating the question in its exact form or varying the words.

The doctor says that the examiners are not called upon to lose time. During the first year I was on the examining board I lost six weeks, besides doing night-work from eight o'clock up to twelve and one o'clock. The compensation was a trifle over three hundred dollars.

I feel glad that the majority of students who come before us are better able to understand the questions than Dr. Trueman; in other words, we do not have many students to examine who criticise the papers and questions as Dr. Trueman has done.

In 1896 the National Association of Dental Examiners did attempt to rule the faculties, and in 1897 the Pennsylvania State

Board withdrew from the National Association, because, as it was then constituted, it could work in harmony with the various colleges of the State. I do not think there is a member of the board who has been active in antagonism against the colleges in the State Society, but that every member of the board has upheld the colleges of the State and their instruction.

The criticism upon the formation of the question, "How do you do so?" I think to be hypercritical. Where there are several ways, the examiner will desire to ask the question, why and how this individual would perform a certain operation or do a certain thing. Of course, the question can be put in a different way to get the same result; but the student understands, and the examiner finds out that he understands, what is meant, and I can see no objection to putting a question in that way.

The difficulty of getting the questions properly printed is a great deal more than any one who has not been through it knows. Apparently you cannot trust the public printer or his office. It is very difficult to get type-written copies of a sufficient number of questions without having grave errors in the type-writing. The last two sets of questions were printed by taking possession of a printer's entire outfit, and standing over the compositor while he put the questions in type, printed them, and destroyed the proofs of his prints and distributed the type. Neither is it easy for one or two persons to control the wording or punctuation of a set of questions that another man may give. No one can appreciate the difficulties that an examining board has to encounter until he has been through it.

I was glad to hear the doctor's paper, glad of his criticisms; it will do good, but I think he has been unjust.

*Dr. Cryer.*—I would like to have heard from Dr. Huey as a member of the Pennsylvania Examining Board. The members of this board were attacked very bitterly as to ignorance and otherwise. I do not remember the exact wording. For my own part, I do not like these questions. I would not like to be examined on that set of questions before us. I do not think they are fair. At the same time, I believe our own board to be honest, fair-minded, and just. They are in harmony with the teachers of the different institutions of our stated society meetings. In his criticism of questions I think he is right. Questions should be plainly put, so that students may understand.

*Dr. S. H. Guilford.*—I admire Dr. Trueman's industry and self-sacrifice as displayed in the preparation of this paper. He has not only given us a very full explanation of the subject, but he has treated it, as he usually does, in a very full and satisfactory manner. There is but one thing I would criticise, and that is the implication of antagonism between the colleges and the State board. There is really no antagonism of that kind. The colleges, so far as I am acquainted with them, are in perfect sympathy with the State board, and the State board has been perfectly just, upright, and cordial with the colleges. The colleges, however, feel that when some of their students come back from the examinations bringing with them the papers containing the questions and ask what such and such a question means, and we have to say we do not know, because the question is so obscure, we do have the feeling that the students have not been asked questions in a proper way.

The matter to-night is not a question as to the integrity of the State board, nor as to any member of it, nor as to the motive back of it. Nobody doubts the power of any State to exercise its power or its care over the people of that State. The same State looks after the health of its citizens, sees that they are supplied with pure food, and that they are not subjected to the influences of any diseases that can be prevented. The establishment of a quarantine station is perfectly right, and to supervise the educational work of the State is right. The only thing is, How should that duty be exercised? The colleges, if they do their duty, have nothing to fear from the examining boards, no matter where they are. If the students are properly trained, they ought to be able to go before the examining board, answer questions, and pass with credit.

There is no question in regard to the fairness of the board. It is simply a question, How shall the State exercise proper superintendence over the welfare of the people? At the present time the best plan seems to be the establishment of dental examining boards. If it prove successful, it will probably be continued. If it happen to fall short or be unsatisfactory, it will have to give way to something better. The work is going on satisfactorily in every way; but, as I said before, there are certain objections to the way in which the examinations are conducted. It is too large a question to go into to-night. Excellent men have been selected in our State, and they are doing their full duty. Whether the present spirit of good-will between the board and the colleges will be kept up, or whether in the course of time political influence may creep in and create antagonism, we do not know. If the latter should occur, some new plan will have to be devised to direct the educational work of the State. Still, let us uphold the present law and give it a good, fair trial. If it has merit, it will stand; if it has not merit, it will fall.

It is hardly necessary to add to the list of questions that have been presented. I have a number here that I have gathered, but the hour is too late to read them.

The trouble with the examination questions, as Dr. Trueman has said, is this: Very frequently they are framed from the standpoint of the individual's own experience. They are not broad enough. A practitioner will have a certain way of performing an operation, or believe that a certain course is the right one to pursue, and will ask a question to bring out that idea, which may not be held by many others. It is simply due to the fact that the gentleman has not been broadened by reading, culture, and experience. When individuals who are not men of intelligence and culture do get on the board, and they frame questions in such a way that they are hard to be understood, it makes the college professor feel a little sore, because he feels that the student has not been justly dealt with. It seems a little anomalous that the same law which requires that the student shall have a fair English preliminary education, and that he shall pursue a college course for three years, should require that he shall after graduation come up for examination before men who in some cases have not had even a grammar-school education, and who, in the framing of questions, violate more rules of rhetoric than would a fourth-grade grammar student.

*Dr. Darby.*—The President has asked me if I could not pour a little oil on the troubled waters. I do not think any is needed. I have been greatly amused, because every man who has spoken was afraid that he had said too much or not enough. I believe Dr. Trueman has done just what he thinks is right, and I believe that the examining board has done just what it thinks is right. I do not think that Dr. Trueman criticised the examining boards as a whole, but he did criticise, and I think justly, the questions that some of these examining boards have put. It is absurd to suppose that any man could answer some of the questions as shown by Dr. Trueman; and when Dr. Roberts said that questions are put, and then, because they are not answered, another question is put to draw out the question asked, he is in error, because all these questions are printed and submitted to the student. If I were examining a candidate there orally, and asked a question that he did not comprehend, I might put that question in a different way, but if I submitted to him a printed examination, and he submitted a written answer, that would end it for that examination, would it not? I ask for information.

*Dr. Roberts.*—Yes.

*Dr. Darby.*—I have no desire—far from it—to criticise the examining boards. There may be incompetent men on the examining boards of all States. So far as my own knowledge goes, the State of Pennsylvania is fortunate in having competent men on its examining board. I believe it is honest, and I do not believe that the colleges are at variance with it; but it is quite possible that a candidate cannot answer a question correctly because he cannot understand the question. It is not always an easy matter to ask a question that every candidate will comprehend, even though it may be an intelligent question and grammatically formed; but when you put to a man questions that have no sense, and then expect him to give you an intelligent answer, the examining board is at fault and not the candidate. We should judge an examining board as it deserves, but not criticise disparagingly the student because he cannot answer questions that are illogical.

*Dr. Roberts.*—I would like to explain, as I have apparently given a wrong impression as to the evolution of the question. If a question is put in one examination and the examiner desires to bring out a certain point in the answer by the student, and he finds that the majority of his answers do not bring that out, but that the students have taken another meaning, he will repeat the question at a subsequent examination, in a different form,—that is, if he have the same object in view that he had at first. If they have answered it according to their understanding, they are marked as they understand it, not necessarily as to whether it is the expected answer, but whether it fits their interpretation of the question.

*Dr. Kirk.*—I would like to say one word more. What I had to say in the beginning was with the desire to see fair play in this matter. We are inclined, I think, to take a great deal of pleasure in getting at the weaknesses of our fellow-men. See at what a tremendous disadvantage the examining boards are placed in a case of this kind. The examinations by dental educational institutions are not matters of public record, while those of the dental board are so by law. My plea is that we should investigate both sides. Let us not attempt to pick the mote out of our neighbor's eye until we have done something with our own little beam.

*Dr. James Truman.*—I did not want to say anything on this subject to-night, but I cannot let the matter drop, late as it is, without placing myself on record in a different way from what has been stated by my colleagues connected with dental education. I am not in harmony with the State boards, nor have I been for many years. The subject is too large a one to enter upon. I simply wish to make that assertion, without arguing the question. I do want to say, however, this much, that criticising questions is not my way of treating the subject, and so far I am in opposition to my friend, Dr. William H. Trueman. Some years ago the State boards began this kind of work. It started in New Jersey, and one of the members of that board travelled over portions of the United States giving the answers of the students, and tried to make them ridiculous. Now, we have the opposite side to-night, but nothing quite equal to that made by the member of the New Jersey State board.

Of course, we know that there are defective questions both in the colleges and the State boards, and more likely in the case of the State board than the colleges, but there is no class of men absolutely free from error in that direction.

There is a deeper meaning beyond this, to my mind, that has never as yet been answered. Are they doing any good work for the general public? I contend that they are not, that they are not preventing incompetent men from practising. Take our own State. Has there been, in the last half-dozen years, any man brought up before the courts for practising without a license? If there has been, I do not know it. It was not done by the State board, by individuals not members of the State board, by this organization, nor by the State Society. Consequently any individual, so far as I know, could commence practice without any license from the State board. Is that protecting the people? Certainly not, as I view it. As it is at present organized, the whole thing is truly a farce.

What we want to know is, what kind of men we have on State boards. I am perfectly satisfied with the honesty of the Pennsylvania State board, and with their ability, so far as I am competent to judge. Is it the proper method of selection to take a man because he is a member of the State Society of Pennsylvania and place him in the State board, nominating, and then the governor appointing him? We want to know exactly what these men are capable of doing, and until this is done properly we will probably have in general poorly organized State boards. They are not in harmony with the colleges; there can never be any harmony while such a difference exists between their methods of doing things and those of the colleges.

*Dr William H. Trueman.*—I wish, Mr. President and gentlemen, to state distinctly and emphatically that I am not opposed, *per se,* to State dental examining boards. Our country is large, and naturally has within its bounds communities that differ widely on educational, as on other matters. We have dental colleges scattered all over the land, and, as is right and proper, these colleges cater to the needs of the communities they serve and from which they draw their support. It is, I take it, practically impossible, and perhaps undesirable, that they should all have the same course of study and the same standard. All of these colleges doing honest work are justly entitled to be considered "reputable," and their diplomas, as diplomas, are equally entitled to respect, notwithstanding that their standards may widely differ.

Now, it is the right of the State to make its own standard, and to say whether or not it is satisfied with a diploma from a dental college, regardless of its source, or to regard a diploma as a certificate entitling its holder to an examination only, or to depend exclusively upon its own agents, the State examining board, to determine the applicant's fitness to practise dentistry within the commonwealth. As things are, the States act wisely, I think, in supervising this matter.

I criticise the boards when they go beyond this and forget the purpose for which they were legally ordained. This they have done. They retain and display to a controlling extent the commercial spirit which, as I have endeavored to show in my paper, gradually crowded out the noble motive that first called them into being. Explain it as you will and mask it as you may, there is an antagonism between the examining boards and the educational institutions. In proof of this I refer you to our periodicals and to the reports of our professional societies, local, State, and national; to the numerous articles and reported discussions they contain attacking these institutions from a commercial standpoint. Some declare their sentiments guardedly, others bluntly. Our plain-spoken friend, Dr. Crouse, tersely states his when he says, "There are at present more practitioners of dentistry than can make an honest living," and holds the colleges responsible for this. (*Dental Digest,* vol. iv., June, 1898, p. 435.) Men who hold such views select the men placed on the examining boards; they select men holding like views with themselves, and look to the examining boards to remedy this state of affairs by making the examinations severe. When I say that there is an antagonism between the examining boards and the educational institutions, I do not mean that there is a personal animosity between those composing the boards and those interested in the dental colleges; there is doubtless very little of this. I refer to the spirit which prompted the remarks I have quoted from the ıeport of the Dental Council of this State. It is a reflection on the colleges as uncalled for as it is out of place. I refer to such actions as that of the New Jersey State board in sending out quotations from absurd answers they had received from applicants who had appeared before them, and that of the same board in collecting and exhibiting at dental meetings the failures in laboratory work—in many instances, no doubt, the result of the applicant being compelled to work with unfamiliar and imperfect tools—as specimens indicating the imperfect training students received in dental colleges. I refer, also, to the derogatory remarks applied to dental colleges by members of examining boards. As individuals, the members of the boards may disclaim this, they may not feel it, while at the same time as members of the boards they are doing their share to keep it up. There are in the United States many State boards, and doubtless there are many serving thereon to whom this criticism does not apply; there are others, however, who have placed themselves unmistakably on record, and to these it does.

As I have stated in my paper, whether an examination serves its intended purpose depends upon those who conduct it. Now, I ask you seriously, Can any one, however honest he may be, conduct a proper examination on a subject he does not himself understand? An important part of these dental examinations depends upon the questions. Look at these I have placed before you. Do they indicate that those who wrote them understood the subjects to which they apply? It is no excuse to say that professors in dental colleges make the same blunders. The State examinations are defended on the ground that the college examinations are not thorough. If the State examiners are not better examiners than the college professors, what good purpose do they serve? Permit me to ask, What care is taken to get competent men on examining boards? Is the qualification of the candidate for this position canvassed at the State society? Is any care taken to select educated men, men who are well posted in current dental literature, who are in touch with the instruction given to dental students, and who are familiar with the teaching of our latest text-books?

There has been too much of the star-chamber methods in examining board business, and too little supervision of their work. Do you think for one moment that the author of any of these questions would have so written them, knowing that they would have been placarded and criticised, as they have been to-night, before a prominent dental society? On the other side of the sea the examination questions have been published for years, and the examining and the educating bodies call attention to them, advising students to procure and carefully study them, that they may know the lines upon which they will be examined at the end of their course. I present to you several sets officially published by the College of Surgeons of London, for the use of those intending to apply for membership, or for a license to practise surgery, or dentistry. There is nothing to be ashamed of in these questions. I commend them to your notice. There is no necessity for repeating questions so often that their publication vitiates the examination. Each branch of dental science is broad enough to furnish proper questions for many examinations. The plea that their publication would lead to making the course of instruction a mere "cramming" for the examination will hold good only when the examiner is a mere catechist.

This matter of State dental examinations is, I fear, far too lightly considered. It is the final act of initiation into the profession, and should be conducted with dignity and decorum. The applicant, whether a recent graduate or a practitioner seeking a new field, here meets for the first time, officially, the representatives of the profession in the commonwealth. He is there to be examined. While the examiners are taking his measure, he is quietly taking theirs. While they are deciding whether he is fit to be their associate, he is asking whether they are fit to be his. I tremble for his answer, as he unfolds and carefully scans the examination papers placed before him. Is it an error, gentlemen, to insist that they shall be in wording, in punctuation, in grammatical construction, in scientific accuracy, and in directness, befitting a learned profession? Is it hypercriticism to complain when they are not so? Am I unjust to the examiners in declaring that these carelessly worded questions are a disgrace to the profession, and that they convert what should be a dignified proceeding into a mere farce? I call the attention of the profession to this matter,—that is the purpose of my paper,—and I ask, Are you satisfied with the examinations as they are now conducted? I ask you to compare the results for good and for evil to the profession and to the community. Considering the turmoil they have caused, and their disorganizing influence in our professional societies, if we cannot find men able to conduct them in a more dignified and proper manner than they have been, better by far let the farce end.

Adjourned.

Otto E. Inglis,
*Editor Academy of Stomatology.*

